# Protein methyltransferase inhibitors as precision cancer therapeutics: a decade of discovery

**DOI:** 10.1098/rstb.2017.0080

**Published:** 2018-04-23

**Authors:** Robert A. Copeland

**Affiliations:** Epizyme, Inc., 400 Technology Square, Cambridge, MA 02139, USA

**Keywords:** protein methyltransferases, chromatin modification, epigenetics, cancer, drug discovery, enzyme inhibitors

## Abstract

The protein methyltransferases (PMTs) represent a large class of enzymes that catalyse the methylation of side chain nitrogen atoms of the amino acids lysine or arginine at specific locations along the primary sequence of target proteins. These enzymes play a key role in the spatio-temporal control of gene transcription by performing site-specific methylation of lysine or arginine residues within the histone proteins of chromatin, thus effecting chromatin conformational changes that activate or repress gene transcription. Over the past decade, it has become clear that the dysregulated activity of some PMTs plays an oncogenic role in a number of human cancers. Here we review research of the past decade that has identified specific PMTs as oncogenic drivers of cancers and progress toward the discovery and development of selective, small molecule inhibitors of these enzymes as precision cancer therapeutics.

This article is part of a discussion meeting issue ‘Frontiers in epigenetic chemical biology’.

## 1. Introduction

**Figure 1.**
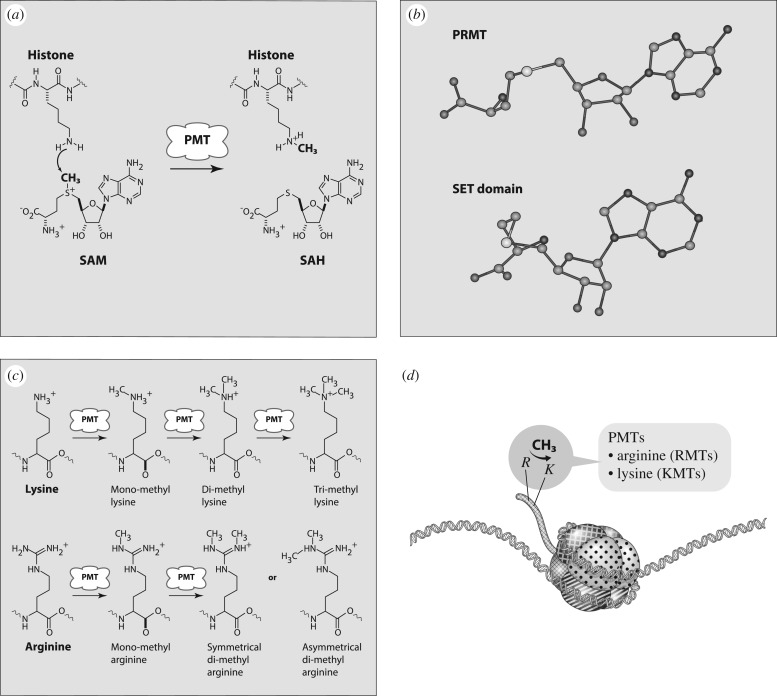
(*a*) Chemical reaction catalysed by PMTs. (*b*) Crystal structures of SAM configuration in PRMTs (top) and SET-domain PKMTs (bottom). (*c*) Methylation states of lysine and arginine. (*d*) Cartoon representing the structure of nucleosomes.

The human protein methyltransferases (PMTs) comprise a large class of enzymes that catalyse the methylation of specific lysine or arginine amino acid side chains within target proteins [1]. All PMTs use a common mechanism of catalysis. The enzymes bind the universal methyl donor *S*-adenosyl-l-methionine (SAM) and the target protein to form a ternary complex. Direct transfer of the methyl group from SAM to the amino acid recipient then ensues, following a classic S_N_2 mechanism, to yield the products *S*-adenosyl-l-homocysteine (SAH) and the methylated protein [1]. The side chain nitrogen of lysine can accept up to three methyl groups and can thus exist in four distinct states of methylation (figure 1).

Likewise, the two side chain nitrogen atoms of arginine can be unmethylated, monomethylated, symmetrically di-methylated or assymmetrically di-methylated. Each of these states of methylation at specific amino acid locations can have distinct conformational consequences for the target protein. In some cases, a single PMT is responsible for multiple rounds of methylation of a specific lysine or arginine location, while in other cases different PMTs perform the distinct, sequential methylation reactions.

Among the known target proteins of PMTs, the histone proteins of chromatin are of paramount interest [1,2]. Chromatin refers to the complexes of histone proteins and chromosomal DNA that form nucleosomes, the fundamental structural unit of chromosomes. Chromosomal DNA is packaged as nucleosomes with intervening stretches of uncomplexed DNA as a mechanism of compacting approximately 2 m of DNA required for the complete human genome into the small volume of a eukaryotic cell nucleus [3]. This highly compacted structure, however, creates steric barriers that restrict access of the transcriptional machinery to promoter regions of genes. Hence, a mechanism is required to relax the compacted structure in a spatio-temporally selective manner with respect to specific gene locations so that transcription may be activated. Conformational control of chromatin structure is enabled by a number of mechanisms working in concert, including methylation of the histone proteins [2]. The amplitude and cadence of these chromatin modifying activities determine the cellular programme of genes to be actively transcribed for any specific cell type at any specific time in the life of the cell. These mechanisms are thus critical to a number of physiological processes, such as cell differentiation and maturation from pluripotent, stem cell-like progenitors.

Around ca. 2008 it became clear that chromatin modifying proteins (CMPs), including examples of PMTs, not only performed critical roles in defining cell identity, differentiation and maturation in normal physiology, but could also play causal roles in human diseases when the activities of specific CMPs were dysregulated [1,4]. In this review, we will focus our attention on the progress made over the past decade to understand the role of dysregulated PMTs in human cancers and efforts towards the discovery and development of selective, small molecule inhibitors of these enzymes as a basis for precision cancer therapeutics.

## 2. Protein methyltransferase as a target class for drug discovery

**Figure 2.**
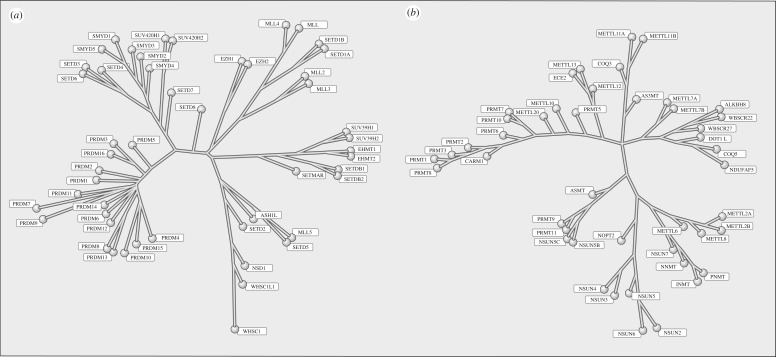
Phylogenic trees for the human PKMTs (*a*) and PRMTs (*b*).

To exploit fully the PMTs as a drug target class requires an understanding of the constituency of PMTs within humans and the structural relatedness of these enzymes to one another. In 2011, Richon *et al.* [5] approached this question by focusing on amino acid sequences that formed the SAM binding pocket of proteins with experimentally verified enzymatic activity as either protein lysine methyltransferases (PKMTs) or protein arginine methyltransferases (PRMTs). In this manner, they set out to derive a single dendogram or family tree that would relate all human PMTs to one another on the basis of similarity of SAM binding pocket sequence. However, these efforts proved fruitless, as there was no way to encompass both PKMTs and PRMTs into a single phylogenic tree. Instead, the PMT class bifurcated into two distinct families. With the exception of DOT1 L, all known PKMTs contain a catalytic domain of approximately 130 amino acids referred to as the SET domain [1]. The PKMT phylogenic tree that resulted from computational survey of the human genome (figure 2) captured all known SET-domain PKMTs and also incorporated a branch containing 16 proteins with a related domain structure known as PRDMs; at least three members of the PRDM branch have been experimentally demonstrated to catalyse protein lysine methylation [6]. The single PKMT lacking a SET domain, DOT1 L, could not be integrated into the PKMT family tree. Curiously, this known PKMT fits well within the family tree for the PRMTs. The family tree for the PRMTs is also shown in figure 2. Early iterations of this tree incorporated DOT1 L and most, but not all, of the proteins known to have verified PRMT activity. Further iterations of the genomic search sought to bring in the missing, verified PRMTs; doing this also added to the tree proteins that had not previously been annotated as PRMTs. These additional proteins represented the METTL and NSUN protein groups, which are annotated in the literature as RNA methyltransferases. We [5] and others [7] demonstrated that at least some of these proteins were capable of methylating protein substrates as well as RNA substrates. Whether or not the PMT activity of the METTL and NSUN proteins is physiologically meaningful remains to be determined. Nevertheless, from a chemical biology and drug discovery perspective, the structural relatedness of the SAM binding pockets of these proteins to those found in bona vide PRMTs must be taken into consideration in terms of ligand selectivity analysis [5].

The understanding of enzyme relatedness that can be gleaned from family trees like those shown in figure 2 can be critical for evaluating ligand selectivity within a protein family. To illustrate this for the PMTs, Richon *et al*. [5] surveyed ligand affinity across representative examples of PKMTs and PRMTs for three nonselective ligands of the PMTs: the universal methyl donor SAM, the universal product of PMT activity, SAH, and a natural product aminonucleoside inhibitor, sinefungin. The affinity for each ligand was mapped upon the PKMT and PRMT family trees and provided clear understandings of variations in specific ligand affinity for different enzymes within the PKMT and PRMT families [5].

## 3. Dysregulation of protein methyltransferase activity in cancer

A large number of PMTs have been implicated as drivers of human cancers due to various genetic mechanisms of dysregulation, ranging from gene amplification to activating mutations within the catalytic domain of the enzyme [2,8–10]. As these have been reviewed extensively in the recent literature, we will not attempt to provide a comprehensive listing of altered PMTs here. What are most attractive from a therapeutic intervention perspective, are genetic alterations that establish a unique dependency of cancer cells on the activity of a specific PMT—a dependency that is not shared by normal cells. This situation affords the greatest opportunity for a significant therapeutic index; that is, a significant difference between the dose of drug required for therapeutic efficacy and that which might cause some adverse safety event. Below we exemplify four mechanism of PMT dysregulation in human cancers that offer this possibility.

### (a) Ectopic localization of protein methyltransferase activity

In normal physiology, a PMT may catalyse methylation of a histone amino acid site at specific gene locations, resulting either in transcriptional activation or repression. If the same enzyme were to instead act at the wrong gene locations with the attendant impact on transcription of those genes, these changes in transcriptional programming could easily result in disease. Such is the case for the enzyme DOT1 L in a form of acute leukaemia known as MLL-rearranged (MLL-r) leukaemia [11]. DOT1 L catalyses methylation of histone H3, lysine 79 (H3K79), leading to activation of gene transcription. This enzyme is known to form complexes with other proteins of the AF and ENL families and these complexes may play a role in homing of DOT1 L enzymatic activity to specific gene locations. MLL-r leukaemia is a relatively rare form of acute leukaemia with a particularly poor prognosis. A universal hallmark of MLL-r leukaemia is the presence of a chromosomal rearrangement affecting the 11q23 locus; indeed, the presence of the chromosomal rearrangement, determined by split gene FISH assays, is used as a diagnostic indicator of MLL-r leukaemia [12]. The 11q23 locus encodes another PMT known as MLL1 (or KMT2A), which normally catalyses the methylation of H3K4 at specific gene locations; these gene locations are determined by the N-terminal region of the MLL1 protein and the partner proteins with which it forms complexes. The catalytic SET domain of MLL1 is located near the C-terminus of the protein. Chromosomal rearrangements associated with MLL-r leukaemia result in fusion proteins involving MLL1. In these fusion proteins the C-terminal SET domain is lost and the remainder of MLL1 is fused to any of a number of protein partners of the AF and ENL families. The ability of the fusion partner proteins to bind and form complexes with DOT1 L brings this enzyme into proximity with gene locations that are normally modified by the action of wild-type MLL1, including the HOXA family of pro-leukaemic genes. Recruitment of DOT1 L to these ectopic gene locations, by complex formation with the MLL-fusion protein(s), results in H3K79 methylation and thus aberrant transcriptional activation of these genes. Hence, the ectopic enzymatic activity of DOT1 L is thought to be leukaemogenic in MLL-r leukaemia, an inference that is supported by preclinical studies of DOT1 L knockdown with shRNA [13] or inhibition by small molecule inhibitors [14,15] and by the clinical activity of the DOT1 L inhibitor pinometostat in MLL-r leukaemia patients (vide infra) [16].

### (b) Chromosomal rearrangements of protein methyltransferases

The example of DOT1 L in MLL-r leukaemia involves an indirect role of a chromosomal rearrangement affecting the activity of a PMT. There are also examples of chromosomal rearrangements that directly affect PMTs and result in pathogenesis. One of the best documented examples of this is the chromosomal rearrangement involving the enzyme NSD2 in t(4;14) multiple myeloma [17]. NSD2 methylates the H3K36 site, resulting in transcriptional activation of affected genes. Approximately 30% of multiple myeloma patients present with a chromosomal rearrangement involving t(4;14). The 4p16.3 breakpoint occurs within a 110 kB region between the genes for NSD2 and FGFR3. The other involved breakpoint, 14q32, occurs within the IgH switch region. The chromosomal rearrangement results in increased expression of FGFR3 under the influence of the IgH Ea enhancer and high level expression of an IgH-NSD2 fusion protein under the influence of the IgH Eμ enhancer. All t(4;14) multiple myeloma patients express the IgH-NSD2 fusion protein while about 30% of these patients lack expression of FGFR3. Hence, the elevated expression of the IgH-NSD2 fusion protein has been implicated as oncogenic in t(4;14) multiple myeloma. Indeed cell lines expressing this fusion protein display significantly elevated levels of global H3K36me2, as expected for elevated expression of a catalytically active NSD2 fusion protein, and shRNA knockdown of NSD2 in t(4;14) multiple myeloma cell lines results in an antiproliferative phenotype, strongly suggesting that therapeutic targeting of NSD2 in the context of the t(4;14) fusion protein may be an effective means of treating this subset of multiple myeloma.

### (c) Hot spot change-of-function mutations within protein methyltransferases

Tri-methylation of the H3K27 site results in strong transcriptional repression that plays a key role in normal B-cell maturation and has also been demonstrated to be oncogenic in several human cancers. This site is methylated in humans exclusively by the polycomb repressive complex 2 (PRC2), a multiprotein complex containing EZH2, or the closely related EZH1, as the catalytic subunit for methyltransferase activity. Elevated levels of H3K27me3, implicated in oncogenesis, have been associated with amplification of EZH2 and other PRC2 subunits [18]. In 2010, an additional mechanism of EZH2 dysregulation in cancer was reported [19]. Morin *et al.* [19] reported the occurrence of point mutations at tyrosine 641 (subsequently designated tyrosine 646) within the SET domain of EZH2 in approximately 20% of germinal centre diffused large B-cell lymphoma (DLBCL) and follicular lymphoma (FL) patients. Somatic mutations of Y641 to histidine, phenylalanine, serine and asparagine were identified in these non-Hodgkin's lymphoma (NHL) patients and a Y641C mutation was also identified in patients with myeloid dysplastic syndrome [20]; subsequent studies also identified A687V and A677G mutations in NHL patients [21,22]. In the initial report by Morin *et al.* [19], the patients bearing mutations were always found to be heterozygous and expressed approximately equal amounts of wild-type and mutant EZH2. This report also compared the enzymatic activity of recombinant wild-type and mutant EZH2 forms in the context of recombinant PRC2 complexes and suggested that the lymphoma-associated mutants were all catalytically inactive. More detailed biochemical studies of the mutant forms of EZH2 demonstrated a novel mechanism of pathogenesis [23]. These studies showed that the wild-type enzyme was most efficient at catalysing the first methylation reaction at H3K27, moderately efficient at catalysing the second methylation reaction and relatively weak at catalysing the third methylation reaction leading to the oncogenic H3K27me3 state. In striking contrast, the lymphoma-associated mutant enzymes were all essentially incapable of catalysing the first methylation reaction at H3K27. They were about equal to wild-type EZH2 in catalysing the second methylation reaction, but the mutant enzymes were far superior to wild-type in catalysing the final methylation reaction leading to H3K27me3. Thus, Sneeringer *et al.* [23] hypothesized that lymphomagenesis in mutant-bearing NHL required the coupled activities of the wild-type and mutant enzymes acting in concert. The wild-type EZH2 performs the first methylation reaction, both wild-type and mutant enzymes perform the second methylation reaction and the superior activity of the mutant enzymes drives high levels of the H3K27me3 state. Consistent with this hypothesis, western blotting of NHL cells in culture showed that cells homozygous for wild-type EZH2 displayed a predominance of the H3K27me2 state, while for cells that were heterozygous for one of the mutant EZH2 forms the predominant methylated state of H3K27 was trimethylated [23,24]. Subsequently, Swalm *et al.* [25] confirmed reaction coupling between wild-type and mutant EZH2 forms in cell-free biochemical assays using recombinant PRC2.

### (d) Synthetic lethal relationships

The term ‘synthetic lethal’ [26], as used today, refers to situations in which loss of function of one gene product confers to a cell a critical dependency on a different gene product, such that knockout or pharmacological inhibition of the second gene product is lethal only to cells with loss of function of the first gene product. This, clearly, provides a mechanism for a large therapeutic index if pharmacological inhibition of a target is only lethal in the context of loss of function of a second protein distinct from the molecular target of drug interaction. Among the PMTs, examples of synthetic lethal relationship occur in various cancer forms. An example from the recent literature involving the PRC2 complex is illustrative of this phenomenon.

The SWI/SNF complex is a multiprotein DNA helicase that functions to modify the topological contacts between chromosomal DNA and histone proteins of nucelosomes, thereby exposing promoter regions of genes as a means of facilitating transcription. It is well known that the activity of the SWI/SNF complex works in direct opposition to the transcriptional silencing caused by PRC2 catalysed H3K27me3; the two reactions are said to be antagonistic to one another. Loss-of-function mutation of different SWI/SNF complex subunits is a common occurrence in human cancers [27]. For example, the INI1 subunit is lost in a number of soft tissue sarcomas, such as malignant rhabdoid tumours (MRTs). MRT is an aggressive cancer that is known to be resistant to chemotherapeutic intervention and is largely treated by surgical resection and radiation treatment [28]. INI1 negativity, as assessed by routine immunohistochemistry, is a diagnostic hallmark of MRT and other INI1-negative cancers. The antagonism between SWI/SNF and PRC2 led some workers to hypothesize that relief of this antagonism in INI1-negative cancers might lead to an over-dependence on PRC2 activity and thereby make these tumours particularly vulnerable to inhibitors of EZH2 [29]. Indeed, shRNA knockdown of EZH2 caused an antiproliferative phenotype in MRT cells in culture [29]. Likewise, inhibition of EZH2 with the investigational drug tazemetostat (EPZ-6438) resulted in potent cell killing of MRT cells in culture, significant tumour growth inhibition in mouse xenograft models of MRT [30] and objective responses among MRT patients in a phase 1 clinical trial of the drug [31].

## 4. Small molecule inhibitors of protein methyltransferases

The past decade has seen significant progress made in the discovery and optimization of small molecule inhibitors of a number of cancer-associated PMTs. For our purposes we will focus attention on compounds that are well-behaved inhibitors of specific PMTs that act by binding to the target enzyme and thereby inhibiting catalytic activity. Table 1 presents examples of such compounds that have been reported for 17 different PMT targets. Within table 1 are examples of compounds that bind in the SAM binding pocket of PMTs and behave as SAM competitive inhibitors. Additionally, compounds that inhibit PMTs have been demonstrated to bind in a variety of pockets on these enzymes and to display a range of biochemical inhibition modalities, as described next.

**Table 1.** Examples of PMT inhibitors. Examples used here are the most advanced compounds presented in the literature, to date, for which the chemical structures have been revealed.

target PMT	most advanced example compound	chemical structure	binding modality	references
DOTI L	pinometostat (EPZ-5676)	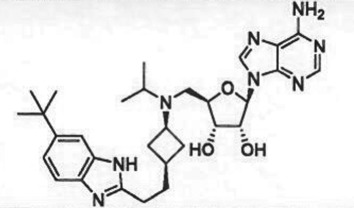	SAM-competitive	[15]
EZH2	tazemetostat (EPZ-6438)	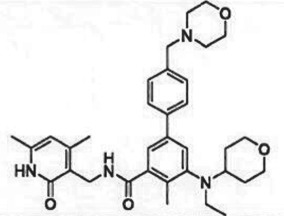	SAM-competitive	[32]
EZH2/1	899145	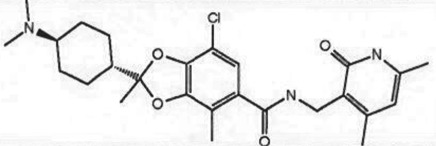	SAM-competitive	[33]
PRC2 complex	SAH-EZH2 (stable peptide)	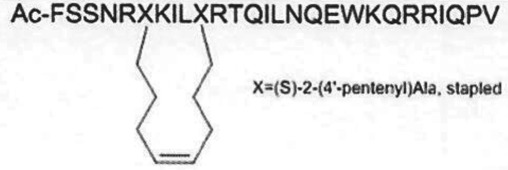	disruption of subunit interactions	[34]
PRC2 complex	A-395	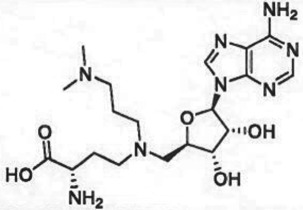	disruption of subunit interactions	[35]
MLL complex	MI-503	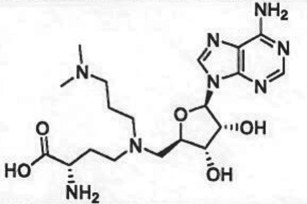	disruption of subunit interactions	[36]
EHMT1/EHMT2	UNC0642	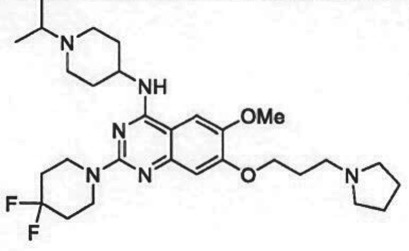	peptide competitive	[37]
SUV420H1/2	A-196	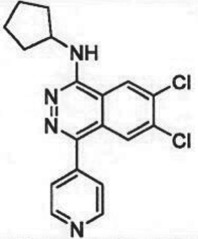	peptide competitive	[38]
SMYD2	EPZ033294	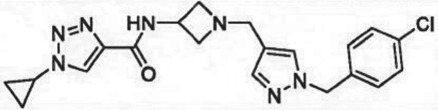	peptide competitive	[39]
SMYD3	EPZ031686	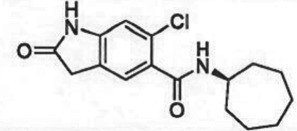	peptide competitive	[40]
SETD7	(*R*)PFI-2	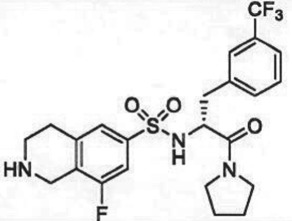	peptide competitive	[41]
SETD8	UNC0379	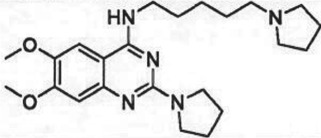	peptide competitive	[42]
pan-type 1 PRMT	MS023	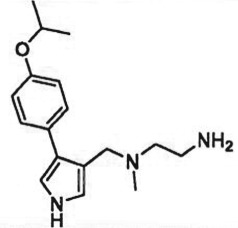	peptide competitive	[43]
PRMT3	SGC707	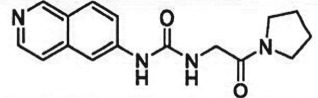	allosteric inhibitor	[44]
CARM1 (PRMT4)	SGC2085	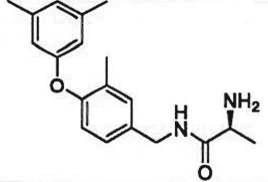	peptide competitive	[45]
PRMT5	EPZ015666	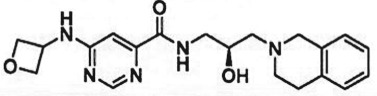	peptide competitive	[46]
PRMT6	EPZ020411	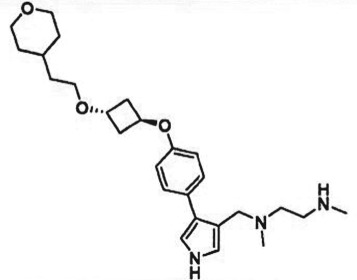	peptide competitive	[47]

### (a) *S*-adenosyl-l-methionine-competitive inhibitors

Potent, selective, cell-permeable, SAM-competitive inhibitors have been reported for a number of PMTs. As summarized in table 1, SAM-competitive inhibitors for the enzymes DOT1 L, EZH2 and EZH1 have been reported and all of these have advanced to clinical testing (see next section). A challenge for SAM-competitive inhibitors is the competition that these will face in the presence of high (approx. 20–40 µM) intracellular concentrations of SAM. Indeed, for many of these SAM-competitive inhibitors one observes a shift in potency from cell-free biochemical assays of enzymatic activity to intracellular assays of methyl mark inhibition, as would be expected for this inhibition modality. Despite this shift in potency, one can routinely observe concentration-dependent inhibition of the appropriate intracellular histone (or other target protein) methyl mark with correlative antiproliferative activity in specific cancer cells.

### (b) Peptide-site binding inhibitors

A number of PMT inhibitors have been reported to bind within the lysine channel of PKMTs or the arginine channel of PRMTs. Two of the first examples of this to be reported were the dual EHMT1/EHMT2 (also referred to as G9a/GLP) compounds BIX01294 [48] and UNC0321 [49]. Subsequent optimization led to UNC0642, a selective, 15 nM inhibitor of EHMT1/2 with good cell permeability and pharmacokinetic properties (table 1). These inhibitors were all shown to be competitive with peptide substrate and noncompetitive with SAM. A ternary complex crystal structure of EHMT1-SAH-UNC0642 demonstrated conclusively that the compound bound in the lysine channel of the enzyme [37]. As summarized in table 1, selective, nanomolar inhibitors of a number of other PKMTs and PRMTs have been identified that bind to their target enzymes in the same substrate channel. In some cases these inhibitors are competitive with peptide substrate, and in other cases they are noncompetitive with peptide substrate. This suggests that in some cases significant contributors of peptide binding to the enzyme are located outside of the lysine/arginine channel; in these cases, the small molecule inhibitor binding within the channel is sufficient to block catalysis but not to block peptide binding [50]. As also summarized in table 1, compounds that bind in the lysine/arginine channel may be noncompetitive or uncompetitive with respect to SAM. In the latter cases, crystallographic data indicates that the inhibitor makes direct or indirect (through an intervening water molecule) interactions with the SAM substrate [46].

### (c) Allosteric inhibitors

PRMT3 functions as a homodimer. In 2012 the Structural Genomics Consortium (SGC) reported a small molecule inhibitor that was a ca. 2.5 µM inhibitor of PRMT3 biochemical activity. Kinetic analysis suggested that the compound was noncompetitive with respect to both substrates, SAM and peptide. X-ray crystallography studies demonstrated that the compound bound in a novel pocket at the base of the dimerization arm of one monomer and made contacts with the α-Y segment of the activation helix of the second monomer of the dimer structure. Subsequent optimization of this chemical series led to SGC707, a very potent (IC50 = 31 nM) and selective PRMT3 inhibitor (table 1). These data demonstrate that PRMT3 enzymatic activity can be modulated by compound binding to a distal, allosteric binding pocket. Whether such allosteric binding pockets exist on other PMTs and can be exploited for inhibitor design remain to be determined.

### (d) Complex disrupting inhibitors

Many PMTs are known to function intracellularly as components of multiprotein complexes. In some cases, the integrity of the multiprotein complex is critical to the enzymatic activity of the catalytic subunit. Such is the case for the PRC2 complex, containing either EZH2 or EZH1 as the catalytic subunit. Isolated EZH2 or EZH1 is devoid of H3K27 methylation activity. It is only in the context of a complex with a minimum of three other PRC2 subunits that EZH2/1 catalytic activity is realized. One of the other critical subunits is known as EED. Kim *et al*. [34] made a stabilized alpha-helical peptide based on a region of EZH2 at the putative subunit interface with EED. This stable peptide, referred to as SAH-EZH2, demonstrated concentration-dependent disruption of the EZH2-EED complex and resultant inhibition of enzymatic activity. Treatment of MLL-AF9 leukaemia cells with SAH-EZH2 resulted in growth arrest and monocyte–macrophage differentiation. Based on these and similar studies, the teams at Abbvie [35] and Novartis developed small molecules that bind to EED and disrupt the PRC2 complex; one of these compounds, MAK683 (structure undisclosed), has recently entered phase 1 clinical testing (vide infra).

For other PMT multiprotein complexes, the non-catalytic subunits are expendable for catalytic activity, but can be critical for directing the PMT activity to intended gene locations. An example of this is the complex between MLL1 or MLL fusion proteins with the protein partner menin. Menin makes direct binding interactions with the N-terminal region of MLL or with the MLL-fusion proteins of MLL-r leukaemia. Genetic ablation studies have demonstrated that an intact menin-MLL-fusion protein complex is required for oncogenesis in MLL-r leukaemia. With these data in mind, the group of Grembecka and co-workers designed small molecule menin binders that would disrupt the protein–protein interaction between menin and MLL [36]. This work resulted in the compound MI-503 (table 1), which bound menin with a *K*_d_ of 9.3 nM and demonstrated anti-proliferative activity in MLL-r leukaemia cells in culture and tumour growth inhibition in mouse xenograft models. In partnership with the biotechnology company Kura Oncology, an optimized compound from the MI-503 pharmacophore series, KO-539 (structure as yet undisclosed), is being advanced into preclinical safety studies in anticipation of clinical testing.

### (e) The role of conformational dynamics in inhibitor binding

**Figure 3.**
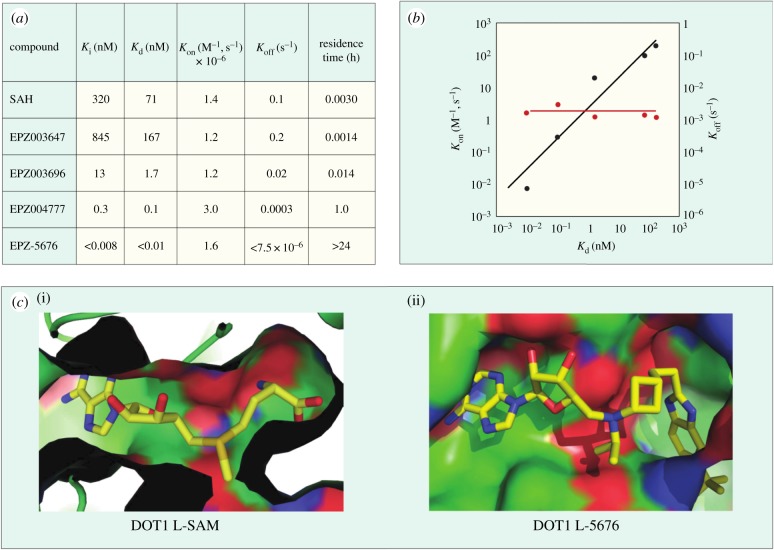
(*a*) Kinetic and thermodynamic values for a series of aminonucleoside inhibitors of DOT1 L. (*b*) Plots of association (*k*_on_) and dissociation (*k*_off_) rate constants for a series of aminonucleoside inhibitors of DOT1 L as functions of the inhibition constant *K*_i_. Note the invariance of the *k*_on_ value across the series. (*c*) Comparison of the crystal structures of DOT1 L bound by SAM (i) and EPZ-5676 (ii) illustrating the formation and ligand engagement of the neomorphic pocket.

Conformational dynamics seems to play an important role in the enzymatic reaction cycle of PMTs, and crystallographic studies of several PMTs have shown flexible loops within the proteins that fold over to partially occlude the catalytic active site from bulk solvent when ligands are bound. Similarly, conformational dynamics can play an important role in small molecule inhibitor interactions with these enzymes as well. This is particularly true in terms of conformational adaptations in response to inhibitor binding that result in high affinity, long-lived (i.e. long drug–target residence time) inhibitor–enzyme complexes [50,51]. Thus several inhibitors of different PMTs have been shown to exhibit long residence times on their target enzymes, including the EZH2 inhibitors GSK2816126 [52] and tazemetostat (EPZ-6438; A. E. Fernandez-Montalvan 2017, personal communication). One of the better-studied examples of the role of conformational dynamics in PMT inhibitor interactions comes from study of a series of aminonucleoside inhibitors of the enzyme DOT1 L. Starting with the reaction product SAH, Basavapathruni *et al*. [53] designed a series of aminonucleoside inhibitors of DOT1 L, ultimately leading to the clinical drug pinometostat (EPZ-5676). The structure–activity relationship (SAR) for this series of inhibitors was studied by biochemical, biophysical and crystallographic methods. It was noted that for all of these compounds, including the reaction product SAH, the rate constant for inhibitor association with DOT1 L was quite slow, ca. 100-fold slower than the expected rate for a diffusion-controlled binding event (figure 3). This led Basavapathruni *et al*. [53] to speculate that some slow conformational change of the enzyme was required for inhibitor binding. Interestingly, the SAR took an abrupt increase in potency when the extended compound EPZ004777 was tested. This compound displayed a *K*_i_ of 300 pM for DOT1 L and an unusually long residence time on the enzyme of ca. 1 h. Further compound optimization led to the clinical candidate pinometostat (EPZ-5676) which displayed even higher binding affinity (*K*_i_ = 80 pM) and longer residence time (greater than 24 h) for DOT1 L. The structural basis for the long residence times of EPZ004777 and EPZ-5676 on DOT1 L was revealed by comparative X-ray crystallography of compounds in this structural series bound to DOT1 L. In the case of SAH and EPZ003696, both inhibitors bound in the well-defined SAM binding pocket with significant overlap of the positions of the substrate SAM and the inhibitors SAH and EPZ003696. In the case of EPZ004777 and EPZ-5676, however, compound binding caused a conformational change of the enzyme active site, creating a neomorphic pocket into which portions of these two inhibitors bound. Interactions with new recognition elements within this neomorphic pocket led to the high affinity and extensive residence times displayed by these compounds (figure 3). In the case of EPZ-5676, the long residence time of this compound appears to lead to durable pharmacodynamics in leukaemia cells. When MLL-r leukaemia cells were treated for four days with EPZ-5676 and the compound was then washed away, an extended lag phase was observed prior to the slow return of intracellular H3K79me2 to control levels. This extended lag period of sustained pharmacodynamics was in part attributed to the long target residence time of the drug [15].

## 5. Clinical studies of protein methyltransferase inhibitors

As summarized in table 2, to date seven PMT inhibitors have entered human clinical trials as investigative cancer therapeutics. Interestingly, this collection of clinical PMT inhibitors spans both PKMT and PRMT targets and represents a range of inhibition modalities, such as SAM-competitive, arginine channel-competitive and complex-disrupting inhibitors.

**Table 2.** PMT inhibitors that have advanced to study in human clinical trials.

compound	target	clinical indication	status	Clinicaltrials.gov identifier
pinometostat (EPZ-5676)	D0T1 L	adults with relapsed/refractory MLL-r leukaemia	phase 1	NCT01684150
pinometostat (EPZ-5676)	D0T1 L	Paediatric patients with relapsed/refractory MLL-r leukaemia	phase 1	NCT02141828
tazemetostat (EPZ-6438)	EZH2	non-Hodgkin's lymphoma	phase 2	NCT01897571
tazemetostat (EPZ-6438)	EZH2	adults with INI1-negative tumours or relapsed/refractory synovial sarcoma	phase 2	NCT02601950
tazemetostat (EPZ-6438)	EZH2	Paediatric subjects with relapsed or refractory INI1-negative tumours or synovial sarcoma	phase 1	NCT02601937
tazemetostat (EPZ-6438)	EZH2	malignant mesothelioma	phase 2	NCT02860286
Tazemetostat (EPZ-6438)/atezolizumab combination	EZH2 + PD-L1	diffuse large B-cell lymphoma	phase 1	NCT02220842
tazemetostat (EPZ-6438)/R-CHOP	EZH2 + various targets	diffuse large B-cell lymphoma	phase 1b/2	NCT02889523
GSK2816126	EZH2	non-Hodgkin's lymphoma, solid tumours and multiple myeloma	phase 1	NCT02082977
CPI-1205	EZH2	B-cell lymphomas	phase 1	NCT02395601
DS-3201b	EZH2/EZH1	non-Hodgkin's lymphoma	phase 1	NCT02732275
MAK683	PRC2 complex disruption	adult patients with advanced malignancies	phase 1	NCT02900651
EPZ015938/GSK3326595	PRMT5	Solid tumours and non-Hodgkin's lymphoma	phase 1	NCT02783300

The first PMT to enter clinical testing was the SAM-competitive DOT1 L inhibitor pinometostat (EPZ-5676). Final reports of the phase 1 studies of this drug in adult and paediatric leukaemia patients have not yet been published. However, an interim report on the adult study was presented by Stein *et al.* [16]. These authors reported that pinometostat demonstrated an acceptable safety profile in adult leukaemia patients and in the target patient population (MLL-r leukaemia patients, vide supra. Clinical activity was demonstrated both in the form of marrow responses and in the form of resolution of leukaemia cutis. The drug is currently being evaluated in combination with other therapeutic modalities for leukaemia and other cancer indications.

Tazemetostat (EPZ-6438), a potent, selective, SAM-competitive inhibitor of EZH2, was the second PMT inhibitor and the first EZH2 inhibitor to enter the clinic. The phase 1 study of this drug was open to all cancer patients. A full report of this phase 1 study has not yet been published, but presentations of interim data from this study were presented in 2015 [54]. Tazemetostat is an orally bioavailable inhibitor of EZH2 and in the phase 1 trial was dosed twice daily (BID) at doses up to 1600 mg. The drug demonstrated an acceptable safety profile and objective responses were observed in non-Hodgkin's lymphoma patients and in solid tumour patients that were histologically negative for either of the SWI/SNF complex subunits INI1 or SMARCA4. Currently, tazemetostat is being tested in phase 2 trials as monotherapy in NHL, relapsed or refractory INI1-negative tumours or synovial sarcoma, mesothelioma, in combination with the standard of care regimen R-CHOP and with the PD-L1 antibody atezolizumab in NHL and in a phase 1 paediatric study in relapsed or refractory INI1-negative tumours or synovial sarcoma (table 2). Subsequent to tazemetostat entering the clinic, a number of other inhibitors targeting the PRC2 complex entered clinical testing. These include the SAM-competitive inhibitors GSK2816126, CPI-1205 and DS-3201b (this last compound inhibits both EZH2 and EZH1 with equal affinity) and the EED-binding complex disrupter MAK683. It will be interesting to see how these various approaches to inhibition of PRC2 activity compare in terms of safety and clinical activity.

The most recent PMT to be targeted for cancer intervention is the protein arginine methyltransferase PRMT5. EPZ015938/GSK3326595 is a peptide competitive, SAM-uncompetitive inhibitor of PRMT5 that was discovered and developed through a collaboration between scientists at Epizyme, Inc. and GlaxoSmithKline. It has recently entered phase 1 clinical testing for solid tumours and NHL.

## 6. Future directions

To date inhibitors targeting three PMTs have advanced to human clinical trials, but this merely represents the vanguard of potential new cancer therapeutics that may derive from continued study of PMTs and inhibitors of these enzymes. Additional studies are already yielding new understandings of the role of dysregulated PMTs, in a variety of human cancers [55]. These studies are also revealing the intricate connectivity among PMTs and related pathways that can result in synthetic lethal relationships, making specific cancer cells vulnerable to selective inhibitors. The continued discovery and development of small molecule inhibitors of these PMTs are thus very likely to result in additional investigative drugs for cancer indications and other disease indications. These efforts will continue to be facilitated by holistic chemical biology and drug discovery efforts, combining detailed biochemistry, cellular and organismal biology, medicinal chemistry and structural biology.

## Data accessibility

This article has no additional data.
